# Analysis of the Influence of the Conduction Sub-Model Formulation on the Modeling of Laser-Induced Incandescence of Diesel Soot Aggregates

**DOI:** 10.3390/e22010021

**Published:** 2019-12-23

**Authors:** Sébastien Menanteau, Romain Lemaire

**Affiliations:** 1 Institut Catholique d’Arts et Métiers, 06 rue Auber, 59016 Lille, France; 2 TFT laboratory, Department of Mechanical Engineering, École de Technologie Supérieure, Montréal, QC H3C 1K3, Canada

**Keywords:** laser-induced incandescence, modeling, conduction, soot aggregate, thermal accommodation coefficient

## Abstract

Laser-induced incandescence (LII) is a powerful diagnostic technique allowing quantifying soot emissions in flames and at the exhaust of combustion systems. It can be advantageously coupled with modeling approaches to infer information on the physical properties of combustion-generated particles (including their size), which implies formulating and solving balance equations accounting for laser-excited soot heating and cooling processes. Properly estimating soot diameter by time-resolved LII (TiRe-LII), nevertheless, requires correctly evaluating the thermal accommodation coefficient $\alpha_{T}$ driving the energy transferred by heat conduction between soot aggregates and their surroundings. To analyze such an aspect, an extensive set of LII signals has been acquired in a Diesel spray flame before being simulated using a refined model built upon expressions accounting for soot heating by absorption, annealing, and oxidation as well as cooling by radiation, sublimation, conduction, and thermionic emission. Within this framework, different conduction sub-models have been tested while a corrective factor allowing the particle aggregate properties to be taken into account has also been considered to simulate the so-called shielding effect. Using a fitting procedure coupling design of experiments and a genetic algorithm-based solver, the implemented model has been parameterized so as to obtain simulated data merging on a single curve with experimentally monitored ones. Eventually, values of the thermal accommodation coefficient have been estimated with each tested conduction sub-model while the influence of the aggregate size on the so-inferred $\alpha_{T}$ has been analyzed.

## 1. Introduction

Understanding the physical-chemical mechanisms leading to the formation of soot particles in combustion processes is of major concern due to the harmful effects on human health and environment associated with the emission of such a particulate pollutant into the atmosphere [1,2]. Doing so, however, implies using and/or developing advanced measurement techniques allowing soot particles to be detected and studied in complex media such as flames or exhaust gases. Among existing diagnostics, laser-induced incandescence (LII) has proven to be a powerful in-situ technique for the characterization of combustion-generated nanoparticles [3,4]. Its principle consists in heating soot up to their incandescing temperature by means of a pulsed-laser excitation source while collecting the subsequent Planck radiation emitted above the ambient flame emission. Since Melton showed that the intensity of the LII signal could be considered as proportional to the soot volume fraction in the investigated medium for detection wavelengths comprised in the visible spectral range [5], this technique has been extensively used and coupled with extinction or cavity ring-down spectroscopy for quantitative measurements of soot concentrations in combustion media [6,7,8]. Since the decay rate of LII signals is representative of the particle cooling process (which itself depends on the particle-specific surface area), LII has also been widely used to infer soot diameter by combining time-resolved detection approaches and signal modeling procedures [9,10,11]. Properly interpreting measured incandescence emissions, however, requires a firm understanding of the physical mechanisms controlling the LII phenomenon. This especially justifies why numerous works have been undertaken during the past few decades to develop theoretical models capable of predicting laser-heated particle radiation [4]. In this field, a majority of authors considered relatively simple model formulations that only incorporate the main phenomena driving the variation of soot internal energy and mass (namely the absorption of the laser energy, the heat conduction between the particles and their surrounding gaseous environment, the radiation, and the sublimation [3,4,5,12,13]. Alternatively, some authors developed more comprehensive formulations of the heat- and mass-balance equations allowing LII signals to be simulated. This is particularly the case of Michelsen who proposed an absorption sub-model accounting for saturation of linear, single-photon, and multi-photon absorption while also considering phenomena such as thermionic emission, soot oxidation, and non-thermal photodesorption of carbon clusters from the particle surface [13,14]. To rule on the consistency of such wide varied model formulations, Lemaire et al. [15] recently proposed a theoretical analysis focusing on the ability of basic and refined LII simulation tools to predict an extensive set of LII time decays and fluence curves obtained by Goulay et al. in a laminar diffusion flame of ethylene [16]. Using inverse techniques to obtain model predictions merging on a single curve with experimental data, Lemaire et al. showed that integrating photolytic mechanisms such as multi-photon absorption and carbon cluster photodesorption was required to reproduce LII signals over a wide range of fluences [15]. While giving insights regarding the physical processes needing to be included in LII models, this work also led to the conclusion that further developments were necessary to account for the effect of aggregate properties on energy and mass transfer phenomena. In addition to the inclusion of additional processes such as annealing, an in-depth analysis of the formulation and parameterization of the sub-models currently used in LII studies to represent absorption and conduction fluxes was eventually pointed out as being of major importance for future developments [15]. In this respect, one should pay particular attention to the rate of energy transferred by heat conduction since this latter directly drives the LII time decays at low-to-intermediate laser fluences, the modeling of which is essential for soot size assessment by time-resolved LII (TiRe-LII). Since heat conduction in LII is expected to occur either in the free-molecular or in the transition regimes (i.e., when the mean free path of the gas molecules is larger or comparable to the soot primary particle size [17]), many authors, therefore, calculate the conductive cooling rate assuming a free-molecular flow [13] while others alternatively consider the approach proposed by McCoy and Cha [18], which is suitable for the transition regime. By analyzing the expression underlying the change of enthalpy of soot particles during conductive cooling, Michelsen et al. proposed an updated sub-model accounting for expansion work that intends to be more consistent for particle/gas systems [19]. That being said, Liu et al. still recommended in their comprehensive review dealing with heat conduction modeling [17] the use of the Fuchs approach [20] that allows covering the entire range of heat transfer regimes from the free-molecular to the continuum ones. Such a brief overview of existing conduction sub-models, thus, illustrates that depicting conductive cooling during laser-induced incandescence of soot is far from being a trivial issue. This is especially true when taking into account the so-called shielding effect that results from the presence of several primary particles within aggregates (such an effect being indeed particularly known to limit the heat conduction between the particles and their surroundings [21]). Omitting the inclusion of such a phenomenon in LII modeling may lead to significant biases in the determination of the soot diameter as well as in the estimation of the thermal accommodation coefficient ($\alpha_{T}$), which is the key parameter for TiRe-LII applications since it determines the probability of a surrounding gas molecule to undergo energy exchange with a soot particle during a collision. To better account for the influence of aggregate properties, Liu et al. proposed a shielding corrective factor [22], whose use led to predictions consistent with those derived from the implementation of the Cercignani-Lampis-Lord (CLL) kernel approach [23], which is assumed to accurately represent the gas-surface scattering physics related to LII experiments. Nevertheless, depending on the selected conduction sub-model and on the inclusion or not of the shielding effect related to aggregates, wide varied theoretical LII signals may be obtained, thus giving rise to important deviations in the soot thermo-physical properties likely to be inferred from fitting and inverse calculation procedures. The present work, therefore, aims to tackle such an issue by simulating a set of LII signals collected in a fully characterized Diesel flame using different conduction sub-models. To do so, a refined LII model built upon a comprehensive version of soot heat- and mass-balance equations has been implemented as described in Section 2. This latter includes an absorption term accounting for saturation of linear, single-photon, and multi-photon absorption processes together with expressions standing for cooling processes by sublimation, conduction, radiation, and thermionic emission in addition to mechanisms depicting soot oxidation and annealing as well as non-thermal photodesorption of carbon clusters. Such a model has been completely parameterized against experimentally monitored data through an original fitting procedure coupling Design of Experiments (DoE) to a genetic algorithm-based solver (see Section 3). Eventually, the influence of the expression used to simulate the conductive cooling process together with the impact of aggregate properties have been analyzed, as detailed in Section 4, to give insights regarding the most suitable modeling option while commenting on the so-derive $\alpha_{T}$ values.

## 2. Methodology

### 2.1. Experimental Approach

**Figure 1:**
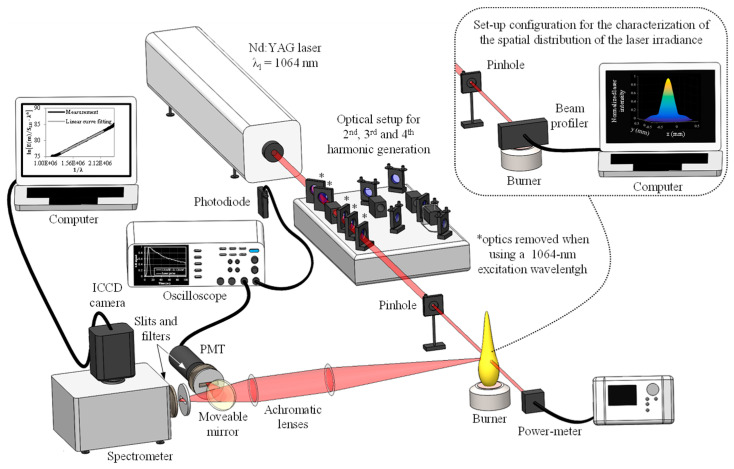
Diagram of the experimental set-up.

The test bench implemented for the purposes of the present work is similar to the one used and extensively described in Reference [24]. It is composed of a flame generation system together with a LII-analytical chain, which are both depicted in Figure 1.

As far as the studied flame is concerned, it is identical to the one previously characterized in References [25,26,27,28,29]. It has been generated using the Lemaire’s spray burner configuration [30], which consists of a McKenna hybrid flat flame burner composed of a 60-mm diameter bronze porous plate with a central 6.35-mm diameter tube allowing the introduction of a direct injection high efficiency nebulizer. A lean premixed methane-air flat flame stabilized on the porous plug has been used to generate the hot gases ensuring the ignition of the low-sulfur Diesel spray exiting from the injector tip. Using the same operating conditions as in Reference [29], a turbulent diffusion flame characterized by a peak soot concentration at 92-mm height above the burner (HAB) has been obtained. Soot samples collected at such a HAB (where a mean temperature of 1850 K has been assessed as detailed below) have, moreover, been analyzed by means of transmission electron microscopy (TEM) and scanning mobility particle sizer (SMPS) analyses [29].

Concerning the LII-analytical chain, it is composed of a Continuum Nd:YAG laser generating pulses at a fundamental wavelength of 1064 nm. As depicted in Figure 1, the central part of the laser beam has been selected using a 1-mm diaphragm and propagated through the flame. By adjusting the distance between the diaphragm and the burner, a beam section of 0.0021 cm^2^ at 1/e^2^ has been obtained at the center of the flame as monitored using a Gentec CCD beam profiler. The laser energy has been continuously measured during experiments by means of a Gentec power detector located behind the flame (the attenuation of the excitation source through the measurement volume being indeed negligible [25]). The time resolved LII signals have been collected perpendicularly to the laser propagation direction using a 300-µm horizontal slit placed in front of a Hamamatsu photomultiplier tube (PMT). Signals have then been digitized and stored by means of a Teledyne Lecroy oscilloscope. Eventually, a Princeton Instruments acton spectrograph has been coupled to a gated ICCD PI-MAX camera for flame temperature assessment through a Planck function fitting procedure [31]. To do so, the calibration of the whole detection chain has been achieved as previously done in References [27,28] using a Gamma Scientific optical sphere noting that the optical setup, as well as the solid angle, has been kept constant during the calibration and the LII measurements. Signals have then been processed following the procedure detailed in References [31,32,33], which led to obtain the above-mentioned temperature value of 1850 K at the investigated peak soot location. 

### 2.2. LII-Model Governing Equations and Resolution Procedure

The model implemented within the present work includes mechanisms accounting for particle heating by absorption of the laser energy (${\dot{Q}}_{abs}$), soot annealing (${\dot{Q}}_{ann}$), and oxidation (${\dot{Q}}_{ox}$) as well as cooling by radiation (${\dot{Q}}_{rad}$), thermionic emission (${\dot{Q}}_{th}$), sublimation (${\dot{Q}}_{sub}$), and conduction (${\dot{Q}}_{cond}$). Based on such processes that are prone to modify the particle energy and mass, one can build a system of coupled differential equations depicting the variations of the soot internal energy rate ($\frac{dU_{int}}{dt}$) and mass ($\frac{dM_{p}}{dt}$) as a function of time following:

(1) $$\{\begin{array}{c} \frac{dU_{int}}{dt}={\dot{Q}}_{abs}+{\dot{Q}}_{ann}+{\dot{Q}}_{ox}-{\dot{Q}}_{rad}-{\dot{Q}}_{th}-{\dot{Q}}_{sub}-{\dot{Q}}_{cond}\\ \frac{dM_{p}}{dt}=\sum_{j=1}^{5}\;{\left(\frac{dM_{p}}{dt}\right)}_{sub,\;j}+{\left(\frac{dM_{p}}{dt}\right)}_{ox} \end{array}$$

where subscripts ‘*sub*’ and ‘*ox*’ denote the contributions of the sublimation and oxidation mechanisms to the mass loss, respectively, while ‘*j*’ stands for the contribution to the particle mass loss of each vaporized carbon cluster C*_j_* (${\dot{Q}}_{sub}$ being computed independently for each carbon cluster from C_1_ to C_5_ following Michelsen [13,14]). More specifically, the rate of change of the energy stored by the particles has been formulated as proposed in Reference [14] to differentiate between the contributions of both the unannealed and annealed fractions of soot whose temperature-dependent densities have been calculated based on linear fits to formulations issued from Reference [34]. Concerning the absorption flux, it has been formulated according to Reference [13] so as to account for the saturation of linear, single-photon, and multi-photon absorption processes. By considering the Rayleigh-Debye-Gans approximation applied to Fractal Aggregates (RDG-FA), the light absorption of soot aggregates can be considered as being equal to the product of the absorption cross section of a single primary particle by the number $N_{p}$ of individual primary particles composing the aggregates. Based on such a theory, the rate of energy absorbed by a soot aggregate can be formulated following the equation below:

(2) $${\dot{Q}}_{abs,r}=N_{p}C_{abs,r}\frac{f_{1,r}\;B_{\lambda 1,r}}{\int_{0}^{t_{l}}q_{exp}\left(t^{\prime }\right)dt^{\prime }}\;\left\{1-exp\left[-\frac{F\;q_{exp}\left(t\right)}{B_{\lambda 1,r}}\right]\right\}+N_{p}\frac{n\;h\;c}{\lambda_{l}}\;k_{\lambda n,r}$$

where the subscript ‘*r*’ stands for either the unannealed and annealed fractions of soot (denoted with subscripts ‘*s*’ and ‘*a*’, respectively, in the following), h corresponds to the Planck constant (6.62 × 10^−34^ J·s), c is the speed of light (2.998 × 10^10^ cm·s^−1^), $f_{1,r}$ and $B_{\lambda 1,r}$ are empirical factors related to the single-photon absorption process (see Section 3), $q_{exp}\left(t\right)$ is the normalized laser irradiance, $t_{l}$ and F stand for the laser pulse duration and energy density, respectively, $\lambda_{l}$ is the laser-excitation wavelength, $k_{\lambda n,r}$ is the rate constant for removal of C_2_ clusters by photodesorption (considering that only C_2_ clusters are produced during the non-thermal sublimation of soot according to [13]), n is the number of photons to be adsorbed to photodesorb C_2_ clusters, and $C_{abs,r}$ corresponds to the absorption cross-section which can be put into equation depending on the soot annealed fraction $X_{a}$ as follows:

(3) $$C_{abs,s}=\left(1-X_{a}\right)\frac{\pi^{2}\;{D_{p}}^{3}}{\lambda_{l}}E\left(m\right)\;and\;C_{abs,a}=X_{a}\frac{\pi^{2}\;{D_{p}}^{3}\;}{\lambda_{l}}f_{a}E_{a}\left(m\right)$$

where $D_{p}$ represents the primary particle diameter while $f_{a}$ is an empirical scaling factor for annealed soot defined in Reference [14]. Regarding the absorption function of soot, its value has been determined through the fitting procedure described in Section 3 for the unannealed particle fraction (E(m)) while it has been set according to the formulation suggested in Reference [14] for the annealed part ($E_{a}\left(m\right)$). Eventually, $k_{\lambda n,r}$ has been calculated following the equation below:

(4) $$k_{\lambda n,r}=X_{s/a}\frac{\lambda_{l}}{n\;h\;c}\;\frac{\sigma_{\lambda n,r}\;\pi \;{D_{p}}^{3}\;N_{sr}}{6}\;\frac{{\left(B_{\lambda n,r}\right)}^{n}}{\int_{0}^{t_{\infty }}{\left[q_{exp}\left(t\right)\right]}^{n}dt}\;\left\{1-exp\left[-{\left(\frac{F\;q_{exp}\left(t\right)}{B_{\lambda n,r}}\right)}^{n}\right]\right\}$$

where $X_{s/a}$ is equal to either $1-X_{a}$ or $X_{a}$ for unannealed and annealed soot fractions, respectively, $\sigma_{\lambda n,r}$ represents the multiphoton absorption cross-section for the photodesorption of C_2_ clusters, $N_{sr}$ is the density of carbon atoms on the surface of primary particles (2.8 × 10^15^ cm^−2^ for unannealed and 3.8 × 10^15^ cm^−2^ for annealed soot [14]), and $B_{\lambda n,r}$ is an empirical saturation coefficient for multiphoton absorption. As far as the annealing (${\dot{Q}}_{ann}$) and oxidation (${\dot{Q}}_{ox}$) fluxes are concerned, they have been implemented as proposed in References [14,19], respectively, while integrating the number of primary particles per aggregate ($N_{p}$) in the expressions accounting for each of these terms. Similarly, $N_{p}$ has been embedded within the governing equations standing for the cooling processes by radiation (${\dot{Q}}_{rad}$) and thermionic emission (${\dot{Q}}_{th}$) that have been expressed as proposed in References [13,14], respectively. Concerning the procedure allowing the sublimation flux (${\dot{Q}}_{sub}$) to be determined, it has been derived from the one extensively presented in Reference [14] even though the rate constants for the photodesorption of C_2_ clusters from unannealed and annealed particles have been estimated based on Equation (4). Eventually, three different conduction sub-models have been implemented for the purposes of the comparative study proposed in Section 4. First, the formulation derived from the work of McCoy and Cha [18] has been considered since this widespread-used formulation directly applies when the heat conduction is expected to occur in the free-molecular regime, which is generally the case in LII studies conducted at atmospheric pressure. According to References [13,15], ${\dot{Q}}_{cond}$ then equates as follows:

(5) $${\dot{Q}}_{cond}=N_{p}\frac{\pi \;{D_{p}}^{2}\;\alpha_{T}\;P_{g}}{R_{p}\;T_{g}}\sqrt{\frac{R_{m}\;T_{g}}{2\;\pi \;W_{a}}}\left[\left(C_{p}-\frac{R}{2}\right)\left(T_{p}-T_{g}\right)\right]$$

where $P_{g}$ is the ambient pressure,$R_{p}$(83.145 bar·cm^3^·mol^−1^·K^−1^) and $R_{m}$(8.3145 × 10^7^ g·cm^2^·mol^−1^·K^−1^·s^−2^) correspond to the universal gas constant expressed in effective pressure and mass units, respectively, $W_{a}$ represents the average molecular weight of air (28.74 g·mol^−1^) considered as a surrogate for flame gases, $C_{p}$ is the molar heat capacity at constant pressure (the expression of which can be found in Reference [13] based on a fit to data from the NIST-JANAF database [35]), R (8.3145 J·mol^−1^·K^−1^) is the universal gas constant while $T_{p}$ and $T_{g}$ stand for the particle and surrounding gas temperatures, respectively. In addition, the updated formulation proposed by Michelsen et al. in [19] has been tested. This latter especially accounts for the expansion work while it considers the integral $\int_{T_{g}}^{T_{p}}C_{p}^{N_{2}}\left(T\right)dT$ instead of the heat capacity $C_{p}$. A Fuchs equivalent sphere modeling approach [20] has finally been implemented as a third sub-model. This latter covers the entire range of heat conduction regimes and allows calculating ${\dot{Q}}_{cond}$ in the free-molecular (FM) and continuum (C) regimes based on Equations (6) and (7), respectively:

(6) $${\dot{Q}}_{cond,FM}=\frac{1}{8}\;\pi \;N_{p}\;\alpha_{T}\;{D_{p}}^{2}\;P_{g}\sqrt{\frac{8\;k_{B}\;T_{\delta }}{\pi \;M_{g}}}\;\frac{\gamma^{*}+1}{\gamma^{*}-1}\left(\frac{T_{p}}{\;T_{\delta }}-1\right)$$

(7) $${\dot{Q}}_{cond,C}=4\;\pi \;N_{p}\left(\frac{D_{p}}{2}+\delta \right)\;\int_{T_{g}}^{T_{\delta }}k_{g}\left(T\right)dT$$

in which $k_{B}$ represents the Boltzmann constant (1.38 × 10^−23^ J·K^−1^), $M_{g}$ denotes the average mass of the gas molecules, $\gamma^{*}$ stands for the mean value of the heat capacity ratio, $k_{g}$ is the heat conduction coefficient of the surrounding gas while δ and $T_{\delta }$ denote the distance, and the temperature related to the limiting sphere separating the free-molecular regime from the continuum one. Eventually, the corrective factor proposed by Liu et al. [22] has also been considered to be integrated within Equations (5) to (7) to analyze the impact of the shielding effect on the modeling of soot LII. In this case, $N_{p}$ has been removed from the above expressions while $D_{p}$ has been replaced by the equivalent sphere diameter $D_{HC}$ whose formulation is defined in Reference [22].

The solving of the system of differential equations specified in Equation (1) leads to infer the variations of the particle temperature $T_{p}$, mass $M_{p}$, and diameter $D_{p}$ (considering spherical primary particles) as a function of space and time. LII signals can then be assessed by integrating the Planck function over the spectral range of the detection system, including its spectral response as previously done and explained in Reference [15]. The normal law derived from the TEM measurements carried out in Reference [29] has been integrated within the calculations so as to represent the size distribution of the primary particles, which is characterized by a mean diameter $D_{p}$ = 16.4 nm and a standard deviation $\sigma_{p}$ = 3.3 at the investigated flame location (i.e., 92 mm HAB). $N_{p}$ has moreover been varied from 1 to 125 (this last value corresponding to the mean number of primary particles per aggregate derived from SMPS measurements [29]) for the purposes of the calculations presented in Section 4 aiming to analyze the effect of the aggregate size on the inferred $\alpha_{T}$ values. The solving of the coupled-differential equations for soot-temperature and mass has been achieved, as done in References [15,36], using MATLAB^®^ software. To do so, the measured spatial distribution of the laser energy has been numerically reproduced and discretized using 17 × 17 elements as validated through a grid-sensitivity analysis. Lastly, the simulated LII signals have been calculated over the entire laser beam and then integrated over the dimensions of the 300-µm slit experimentally used for a proper comparison with measured data.

## 3. Model Parameterization

**Figure 2:**
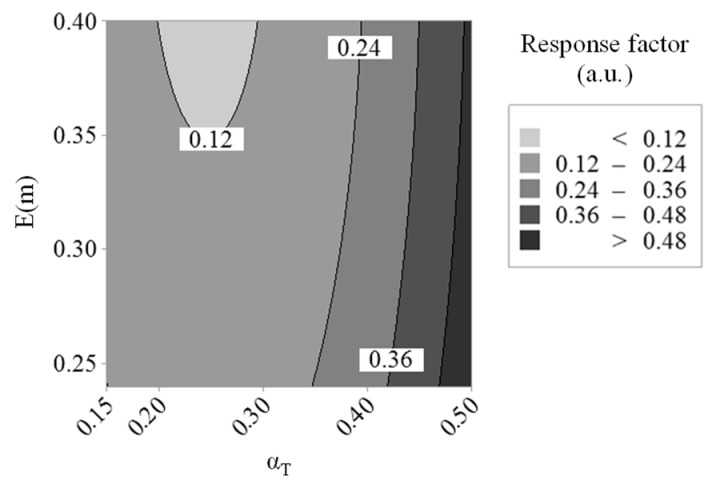
Response surfaces obtained for $\alpha_{T}$ and E(m) using DoE-1 (the response factors plotted on the graph being calculated based on the sum of the root-mean-square deviation between experimental and numerical results).

The values of the different parameters integrated within the governing equations standing for the energy fluxes described in Section 2 are issued from the references reported therein (i.e., Reference [13] for ${\dot{Q}}_{th}$, Reference [14] for ${\dot{Q}}_{ann}$, ${\dot{Q}}_{rad},$ or ${\dot{Q}}_{sub},$ and Reference [19] for ${\dot{Q}}_{ox}$) except for some specific factors whose values are not available in the literature for a 1064-nm laser-excitation wavelength. This includes different parameters involved in the Michelsen absorption and sublimation sub-models that have been developed and validated based on data acquired using a visible excitation wavelength of 532 nm [13,14]. To obtain simulated signals merging on a single curve with measured ones, a parameterization of the model implemented herein has, therefore, been necessary to derive the values of the multiphoton absorption cross-sections for C_2_ photodesorption ($\sigma_{\lambda n,s}$ and $\sigma_{\lambda n,a}$), the empirical saturation coefficients for linear ($B_{\lambda 1,s}$ and $B_{\lambda 1,a}$) and multiphoton absorption ($B_{\lambda n,s}$ and $B_{\lambda n,a}$), the enthalpies required to photodesorb carbon clusters ($\Delta H_{\lambda n,s}$ and $\Delta H_{\lambda n,a}$), the absorption function of unannealed soot E(m) ($E_{a}\left(m\right)$ being issued from the data reported in Reference [14] as mentioned in Section 2.2), and the thermal accommodation coefficient $\alpha_{T}$. Such a number of unknown parameters has still been reduced to six as the present study mainly focuses on the low-to-intermediate fluence regimes (i.e., < 0.2 J·cm^−2^) for which the soot annealed fraction remains low, as verified during the calculations. $\sigma_{\lambda n,a}$, $B_{\lambda 1,a},$ and $B_{\lambda n,s}$ have, therefore, been set equal to $\sigma_{\lambda n,s}$, $B_{\lambda 1,s}$, and $B_{\lambda n,s}$ in a first stage noting that additional simulations performed for fluences as high as 0.34 J·cm^−2^ with such a parameterization still led to satisfactorily reproduce experimentally monitored signals (see the fluence curve reported and discussed below). The value of the enthalpy required to photodesorb carbon clusters from annealed particles $\Delta H_{\lambda n,a}$ has been set as proposed in Reference [14]. Eventually, a free-molecular regime has been considered for the fitting procedure with $N_{p}$ = 1 noting that both the influence of the conduction sub-model and the shielding effect on inferred $\alpha_{T}$ values will be studied more specifically in the next section (the other factors to parameterize being not impacted by the conduction process or $N_{p}$). The fitting of $\sigma_{\lambda n,s}$, $B_{\lambda 1,s}$, $B_{\lambda n,s}$, $\Delta H_{\lambda n,s}$, E(m), and $\alpha_{T}$ has been achieved by means of a two-step optimization procedure. First, DoEs have been performed to minimize an objective function based on the root-mean-square deviation between numerical and experimental LII signals (see Reference [15]) with the aim of narrowing the ranges of expected parameter values while ensuring that obtained results remain physically consistent with available data from the literature, as discussed below. A genetic algorithm has then been applied on the derived limited variation domains to get the final set of parameters allowing the best fit between simulated and measured data to be obtained. As far as DoEs are concerned, a full central composite design [37] has been chosen since it offers a good compromise between accuracy in the obtained multivariate regression and number of design points [38] (noting that 90 simulations have been required to perform the six-factor DoE analyses). In a first step, a series of response surfaces have been plotted by considering the minimization of an objective function based on experimental and numerical LII time decays obtained at three different laser fluences covering the whole range of laser excitations experimentally used. The results issued from the analysis of such a design of experiments (named DoE-1 below) led to obtain an adjusted-R^2^ of ~86% and a predicted-R^2^ of ~75% for the full quadratic model (including the linear, quadratic, and interaction terms) with an analysis of the variance allowing a corresponding Fisher-test *p*-value less than 10^−4^ to be obtained, which indicates that the model can be considered as statistically significant. Based on the *p*-values for the linear, quadratic, and interaction terms used to determine the polynomial regression coefficients, it has been found that $B_{\lambda 1,s}$, $\sigma_{\lambda n,s},$ and $\Delta H_{\lambda n,s}$ were not truly significant considering the default threshold of 0.05 fixed for DoE-1. The obtained response surfaces, therefore, did not allow identifying well-defined optimized values for such parameters, even though it still has been possible to infer a thermal accommodation coefficient of ~0.25, as illustrated in Figure 2. 

**Figure 3:**
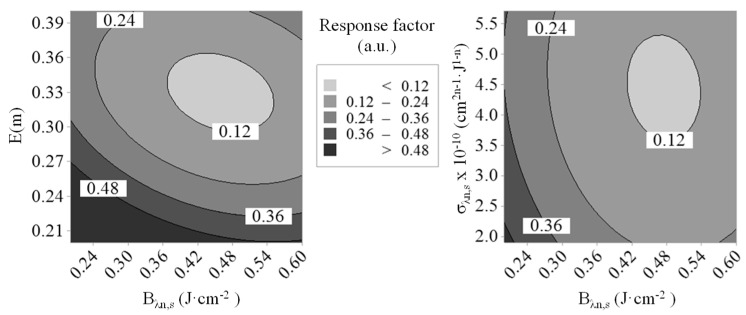
Response surfaces obtained for E(m), $B_{\lambda n,s}$, and $\sigma_{\lambda n,s}$ using DoE-2.

With the view to infer suitable values for E(m), $B_{\lambda 1,s}$, $B_{\lambda n,s},$ and $\sigma_{\lambda n,s}$, which are all factors governing the absorption flux, a second design of experiments (DoE-2) has been established by defining an objective function based on the numerical and experimental fluence curves (noting that both $\alpha_{T}$ and $\Delta H_{\lambda n,s}$ do not influence the laser absorption process, which is especially depicted by means of the fluence curves). Here again, the significance of the model has been concluded based on adjusted-R^2^ and predicted-R^2^ values of ~91% and ~85%, respectively. Obtained response surfaces plotted in Figure 3 then allowed identifying optimal values of ~0.33 for E(m), 0.46 J·cm^−2^ for $B_{\lambda n,s}$, and 4.3 × 10^−10^ cm^2n−1^·J^1−n^ for $\sigma_{\lambda n,s}$.

**Figure 4:**
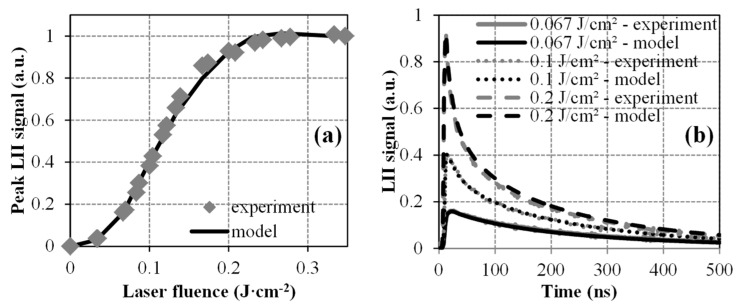
Comparison of simulated and measured LII fluence curves (**a**) and time decays (**b**).

As far as $B_{\lambda 1,s}$ and $\Delta H_{\lambda n,s}$ are concerned, none of the implemented DoEs allowed defining narrower ranges of values with respect to the intervals initially fixed (i.e., between 0.8 and 1.5 J·cm^−2^ for $B_{\lambda 1,s}$ as supported by the experimental results from Reference [39] and between 1 × 10^5^ and 3.4 × 10^5^ J·mol^−1^ for $\Delta H_{\lambda n,s}$ based on the results of a previous optimization work [40]). As a consequence, a genetic algorithm (*ga* function of MATLAB^®^) has been used to refine obtained results and, thus, finalize the parameterization of the LII model implemented in this work. To do so, the above-mentioned ranges of values have been selected for $B_{\lambda 1,s}$ and $\Delta H_{\lambda n,s}$ while intervals of ±15% around the optimum values defined by the DoEs have been fixed for the other parameters in order to constrain the optimization algorithm. The objective function has been set as done for DoE-1 but considering all the time decays acquired for fluences comprised between 0 and 0.34 J·cm^−2^. A population of 20 individuals has been necessary to perform the calculations over around 150 generations to reach a relative tolerance of 10^−4^ for the final solution, which has been found sufficient. Doing so, the following optimized values have been derived: E(m) = 0.29, $\sigma_{\lambda n,s}$ = 4.2 × 10^−10^ cm^2n−1^·J^1−n^, $B_{\lambda 1,s}$ = 1.15 J·cm^−2^, $B_{\lambda n,s}$ = 0.41 J·cm^−2^, $\Delta H_{\lambda n,s}$ = 1.7 × 10^5^ J·mol^−1^, and $\alpha_{T}$ = 0.26. Simulated LII signals calculated using such a parameterization are plotted in Figure 4 and are compared with their experimentally monitored counterparts.

As one can see, numerical results clearly merge on a single curve with measured ones, which thus validates the consistence of the derived parameter values. Such a conclusion is supported by the fact that the estimated soot absorption function is in perfect agreement with the value determined in the same flame and at the same HAB in Reference [29] while the inferred thermal accommodation coefficient is in line with the classical range of values typically met in LII studies (i.e., between 0.23 and 0.37 [4,13]). One can add that identical $\sigma_{\lambda n,s}$, $B_{\lambda 1,s}$, $B_{\lambda n,s}$, $\Delta H_{\lambda n,s},$ and E(m) values have been derived when reapplying the optimization procedure described above but considering $N_{p}$ as different from 1. In such a case, only the thermal accommodation coefficient is required to be changed, as expected and stated above, since this latter represents the only parameter likely to be impacted by the number of primary particles within aggregates through the shielding phenomenon (multi-diffusion within aggregates being not considered herein [41]). That being said and since the consistency of the proposed model has been validated as illustrated in Figure 4, this refined simulation tool has, therefore, been used to analyze the influence of both the conduction sub-model and the aggregate size on the estimated $\alpha_{T}$ values.

## 4. Influence of the Heat Conduction Sub-Model and Aggregate Size on $\alpha_{T}$ Assessment

**Figure 5:**
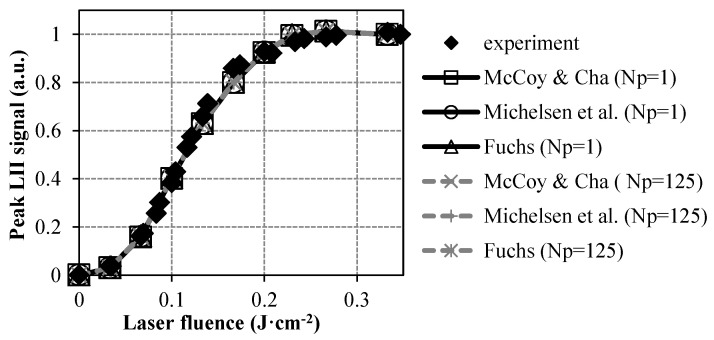
Comparison of measured and simulated LII fluence curves for different conduction sub-models and $N_{p}$ values.

The three conduction sub-models presented in Section 2 (namely, the McCoy and Cha [18], the Michelsen et al. [19], and the Fuchs [20] ones) have been implemented to compare the values of the thermal accommodation coefficient inferred when using such different formulations. Calculations have been performed considering a number of particles per aggregate of 1 in a first stage. The shielding effect has then been taken into account by integrating the corrective factor from Liu et al. [22] based on a mean $N_{p}$ of 125 that corresponds to the value previously determined by SMPS in Reference [29]. As illustrated in Figure 5, LII fluence curves remain logically insensitive to the selected conduction sub-model. Such an observation is actually in line with the fact that the peak of the LII signal is fundamentally driven by the absorption flux while the role played by the conduction cooling process only becomes significant for times much greater than the laser pulse duration.

**Figure 6:**
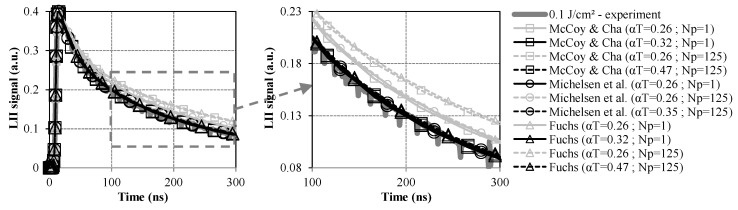
Comparison of measured and simulated LII time decays obtained for a fluence of 0.1 J·cm^−2^ using different conduction sub-models and $N_{p}$ values.

As expected, LII time decays are much more sensitive to the conduction sub-model for their part (see Figure 6). Obtaining simulated signals merging on a single curve with measured data implies deriving different $\alpha_{T}$ values depending on the considered sub-model and on the number of particles per aggregate. In this regard, it has been possible to assess $\alpha_{T}$ of 0.32 and 0.47 for $N_{p}$ = 1 and $N_{p}$ = 125, respectively, when using the McCoy and Cha and the Fuchs modeling approaches. The fact that both models lead to similar results is actually due to the heat conduction process that mainly operates in the free-molecular regime as supported by the calculated Knudsen numbers that are higher than ~30. As a consequence, the formulation of the Fuchs sub-model reduces to an expression similar to the one related to the McCoy and Cha approach. Similar rates of energy dissipation by conduction are, therefore, predicted leading to identical $\alpha_{T}$ values.

Though the $\alpha_{T}$ obtained for $N_{p}$ = 125 is slightly higher than the range of values commonly considered in LII-modeling studies (from 0.23 to 0.37 according to Reference [13]), it still remains consistent with the thermal accommodation coefficients used in References [42,43] while being significantly lower than the value reported in References [10,44]. It, moreover, stays consistent with the data issued from References [12,45] where relatively similar and even higher coefficients were determined by numerical simulations. That being said, different factors may be raised to attempt explaining why the processing of the data gathered herein leads to a somewhat elevated $\alpha_{T}$ as compared to the data reported in Reference [13]. First, the nature of the investigated combustion environment (i.e., a turbulent Diesel flame) significantly diverges from the laminar flames of gaseous fuels usually considered to derive thermal accommodation coefficients (see References [21,46] as well as Reference [4] and references therein). Since $\alpha_{T}$ is known to significantly depend on the chemical structure and molecular mass of the surrounding gases involved in the heat conduction process [47,48], burning wide varied fuels may lead to far different oxidation products prone to influence the value of the thermal accommodation coefficient. In addition, one has to remind that the $\alpha_{T}$ estimated for $N_{p}$ = 125 is issued from calculations integrating the corrective factor proposed by Liu et al. accounting for the shielding effect [22] that especially leads to increase the $\alpha_{T}$ of ~47% with respect to the value determined when considering isolated primary particles (i.e., when $N_{p}$ = 1). Such a trend is actually consistent with the effect of the shielding process that tends to reduce the rate of energy dissipation by conduction per primary particle contained in the aggregates as compared to the conduction flux calculated when neglecting such a phenomenon. Consequently, for a given conductive cooling rate, taking into account the shielding effect intrinsically leads to infer higher $\alpha_{T},$ which justifies why lower thermal accommodation coefficients have often been found in studies conducted without taking into account the aggregate properties, as is the case in Reference [46] for instance. Eventually, the results reported herein also corroborate the analysis made by Kuhlmann et al. [49] who showed that taking into account an effective heat transfer surface for fractal aggregates instead of modeling the heat conduction flux considering isolated primary particles induces a rise of the $\alpha_{T}$ from 0.25 to 0.43 (such values being very close to those determined in the present work). It thus demonstrates the importance of correctly taking into account aggregate properties so as to assess consistent $\alpha_{T}$ values by LII modeling. Lastly, it is worthy to note that the conduction sub-model proposed by Michelsen et al. leads for its part to lower the thermal accommodation coefficient to 0.26 and 0.35 for $N_{p}$ = 1 and 125, respectively. In fact, such a trend, also highlighted in Reference [19], can be related to a higher rate of energy dissipation induced by the inclusion of the expansion work of gas molecules, which tends to lower the so-inferred $\alpha_{T}$ to compensate for such an effect. While leading to a thermal accommodation coefficient falling in the lower range of values currently used in LII modeling studies, the inclusion of the shielding effect does not increase the $\alpha_{T}$ outside of the above-mentioned and commonly assumed 0.23–0.37 limits [4,13]. The use of the conduction sub-model from Michelsen et al. remains rare in LII-modeling studies, however. It is moreover derived from an expression valid for a free-molecular flow regime and is as such less comprehensive than the Fuchs modeling approach, which thus appears to be more suitable to model heat conduction in the context of soot LII over an extended range of conditions (especially for high pressure applications) as concluded by Liu et al. [17].

## 5. Conclusions

The present work pertained to the analysis of different factors influencing the estimation of the thermal accommodation coefficient involved in the conductive cooling process of laser-heated soot. To do so, a series of laser-induced incandescence signals have been acquired within a turbulent diffusion flame of Diesel. A comprehensive formulation of the heat- and mass-balance equations accounting for the LII process has then been implemented and entirely parameterized by means of an advanced optimization approach coupling in an original way DoE and genetic algorithms. Specific attention has been paid to the simulation of the conduction flux through the implementation of three different sub-models while considering a corrective factor accounting for the shielding effect within soot aggregates. Based on obtained results, the following conclusions can be drawn.

- The proposed parameterization procedure allowed inferring values of different soot properties and absorption factors never reported before for a 1064-nm laser excitation wavelength. More particularly, the following results have been obtained: $\sigma_{\lambda n,s}$ = 4.2 × 10^−10^ cm^2n−1^·J^1−n^, $B_{\lambda 1,s}$ = 1.15 J·cm^−2^, $B_{\lambda n,s}$ = 0.41 J·cm^−2^, and $\Delta H_{\lambda n,s}$ = 1.7 × 10^5^ J·mol^−1^. Additional works are still in progress in our lab so as to confirm and/or refine such values using extended datasets.
- The soot absorption function derived herein (i.e., E(m) = 0.29) turned out to be perfectly consistent with the one previously determined in Reference [29] at the same HAB within the same flame, which strengthens the consistency of the proposed modeling approach.
- As far as the estimation of $\alpha_{T}$ is concerned, the use of the McCoy and Cha and Fuchs conduction sub-models led to identical results since the conductive cooling process mainly occurs in the free-molecular regime. On the other hand, the formulation proposed by Michelsen et al. led to inferred $\alpha_{T}$ values ~26% lower, according to the observations and explanations given in Reference [19].
- Eventually, the inclusion of the shielding corrective factor from Liu et al. [22] integrating the measured number of particles per aggregate (125) led to determine $\alpha_{T}$ up to ~47% higher than when considering a $N_{p}$ of 1.
- In conclusion, the present study showed that an $\alpha_{T}$ of 0.47 appears to be well adapted to properly simulate the conductive cooling process of the Diesel soot aggregates that have been analyzed by LII.

While illustrating the importance of correctly taking into account aggregate properties to derive thermal accommodation coefficient values, the present work also gave insights regarding the consistency of the Fuchs conduction sub-model, as previously concluded in Reference [17]. Such a comprehensive modeling approach allowed deriving consistent results (simulated LII time decays indeed merging on a single curve with those measured herein) while being more likely to properly represent the different conduction regimes possibly encountered during LII studies. That being said, additional analyses performed at different HAB in the investigated Diesel flame are currently in progress in our lab so as to complement the present study especially as far as the dependence of E(m) and $\alpha_{T}$ towards the particle maturation stage is concerned.
